# Editorial: Commercialization and industrialization in experimental pharmacology and drug discovery: 2023

**DOI:** 10.3389/fphar.2024.1528987

**Published:** 2024-12-11

**Authors:** Sirajudheen Anwar, Antonio Speciale

**Affiliations:** ^1^ Department of Pharmacology and Toxicology, College of Pharmacy, University of Hail, Ha’il, Saudi Arabia; ^2^ Department of Chemical, Biological, Pharmaceutical and Environmental Sciences, University of Messina, Messina, Italy

**Keywords:** experimental pharmacology, drug discovery, innovative therapeutic agents, natural products, network pharmacology

Innovative therapeutic agents are developed through the dynamic and complex process of new drug discovery, which combines state-of-the-art science and technology. Target identification is usually the first step in the process, during which scientists identify the biological pathways that contribute to the development of disease. Finding these targets has become much easier thanks to developments in proteomics and genetics (Zhang et al., 2020). Natural products offer a wealth of bioactive chemicals with a variety of therapeutic capabilities, making them a longstanding pillar of drug development. By helping researchers comprehend intricate biological connections and mechanisms at a systems level, network pharmacology integration has improved the investigation of these substances. Network pharmacology helps promote a comprehensive approach to drug development by charting the connections among natural products, their biological targets, and related disease pathways (Li et al., 2020; Zhang et al., 2021). Additionally, the manipulation of plant and microbial cells to create complex compounds that would otherwise be difficult to create chemically has also been made easier by developments in biotechnology. This method not only makes it possible to produce medications in a more environmentally friendly and affordable manner, but it also makes it possible to find new treatments that take advantage of these organisms’ special biochemical properties (Wang et al., 2018).

With this in mind, Hussain et al. applied *in vitro* and *in silico* methodology to study the anti-cytotoxic attributes of an anthraquinone, aloe-emodin (AE), against androgen-independent human prostate cancer DU145 cells. It exerted strong cytotoxic effects in these cells, raised lactose dehydrogenase levels, caused oxidative stress mediated by reactive oxygen species (ROS), and activated caspase-3 and -9. Together with their target genes, such as cyclin D1 and c-myc, AE also decreased the survival of mitochondria by raising cytosolic cytochrome-c levels and decreasing Wnt2 and β-catenin mRNA levels. Providing evidence of a potential preclinical source for anticancer treatment.

Another study by Anwar et al. used a variety of computational and network pharmacology methods to investigate the binding dynamics of quinazoline derivatives with EGFR. These methods include 3D-QSAR, ligand-based virtual screening, molecular docking, fingerprinting analysis, ADME, and DFT-based analyses (MESP, HOMO, LUMO), which are complemented by molecular dynamics simulations and MMGB/PBSA free energy calculations. Comprehensive MD simulation studies on three specific ligands—1Q1, 2Q17, and VS1—showed their stability and reliable interaction over a 200 ns time span. Their excellent EGFR1 affinity was highlighted by MM/GBSA values, which also supported their docking scores. With regard to the creation of EGFR1 inhibitors, these findings establish VS1 as a very promising lead, offering important information for creating new, potent EGFR1 inhibitors.

An interesting investigation was conducted employing captopril and pantoprazole as metallo-β-lactamase inhibitors to overcome the resistance of *K. pneumoniae* isolates to carbepenam. The hydrolytic activities of metallo-β-lactamases were decreased by sub-inhibitory quantities of pantoprazole and the well-known metallo-β-lactamase inhibitor captopril, with pantoprazole exhibiting higher inhibiting effects. When used with meropenem, both drugs showed synergistic effects. Additionally, pantoprazole suppressed the production of the metallo-β-lactamase genes blaNDM and blaVIM. To sum up, pantoprazole and meropenem can be used together as a novel treatment approach for severe infections brought on by *K. pneumoniae* that produces metallo-β-lactamases (Abdulaal et al.).

The elevated plus maze, open-field test, novel item identification, and passive avoidance are all indicators of cognitive deficiencies associated with Alzheimer’s disease. In the study by Liu et al., the effect and molecular mechanisms of formononetin to alleviate these symptoms were investigated using Senescence-accelerated mouse-prone 8 (SAMP8) mice as an Alzheimer’s model. Hippocampus RNA analysis was used in the study to analyse RT-qPCR, RNA-seq, along with histopathologic analysis and bioinformatics analysis. Formononetin treatments improved morphological alterations and reduced behavioral deficits, as shown by Nissl and H&E staining. Different patterns of gene expression were found by RNA-seq in SAMP8 and SAMR1 mice. Formononetin either reduced or normalized differentially expressed genes in SAMP8 mice. Neuroinflammation was caused by deficits in the NRF2 and SIRT-1 signaling pathways and increases in proinflammatory markers and immunological dysfunction, according to Ingenuity route Analysis (IPA) of the canonical route and upstream regulators. These molecular alterations were reduced or even reversed by formononetin therapy. Other AD mouse models’ transcriptome profiles from the GEO database were compared to the transcriptome of SAMP8 mice.

As a novel approach, Bendary et al. evaluated the antivirulence properties of sulforaphane at sub-inhibitory concentrations while combing with anti-pseudomonal medications. Furthermore, the virtual affinity of sulforaphane for Quorum sensing (QS) receptors was investigated, and its impact on QS gene expression was measured. Sulforaphane dramatically reduced the production of virulence extracellular enzymes such hemolysins, elastase, and proteases, as well as other virulence factors like pyocyanin, biofilm formation, motility, and resistance to oxidative stress. When used with antipseudomonal drugs, sulforaphane decreased their minimum inhibitory concentrations (MICs), producing synergistic benefits. Sulforaphane exhibits potential as a strong anti-virulence and anti-QS agent that can be used in conjunction with traditional antimicrobials to effectively treat severe illnesses.

β-nicotinamide mononucleotide (NMN) has several uses in food and medication development. Yu et al. summarized these uses in order to provide a thorough and in-depth review of the properties, metabolic pathways, pharmacological effects, and advancements in human clinical trials. The authors depicted the importance of using NMN for antioxidant and anti-aging treatment. Furthermore, findings from recent clinical investigations have demonstrated that oral administration of NMN considerably raised the levels of NAD+ in the blood of both patients and volunteers. Following that, a marked rise in the skeletal muscle insulin signal (Akt and mTOR phosphorylation) was noted in postmenopausal women who were overweight or obese and had prediabetes.

Another review article by Buczyńska et al. suggested that metformin has potential as an adjuvant treatment for a number of diseases because it affects immunological responses, hormone synthesis, and lipid metabolism pathways, all of which can become dysregulated in disease states and lead to elevated oxidative stress. Metformin exhibits antioxidative actions via a variety of molecular pathways, such as downregulating the expression of NADPH oxidase, activating the SIRT3 and AMPK pathways, and restoring paraoxonase activity. Together, these strategies improve the state of oxidative stress and reduce the formation of reactive oxygen species in a variety of cellular contexts and disease models

In summary, this Research Topic collects original research and review articles from academic and pharmaceutical professionals about the development, discovery, commercialization, and industrialization of novel treatments, as well as synthetic and semi-synthetic drugs originated from bacterial, plant, or animal cells, focusing on their biological targets and related disease pathways, and encourages the development of new medicinal substances.
